# Retrograde intra-renal surgery: Where are we today?

**DOI:** 10.4103/0970-1591.44258

**Published:** 2008

**Authors:** Percy Jal Chibber

**Affiliations:** Department of Urology, Jaslok Hospital and Research Centre, 15, G Deshmukh Road, Mumbai-400 026, India

The advent of transurethral surgery in the 1930s marked the birth of Endourology as a specialty, which was to bloom nearly half a century later with the introduction of three new developments, namely, Percutaneous Renal Surgery (PCNL), Ureterorenoscopy (URS) and Shockwave Lithotripsy (SWL). URS initially involved the passage of rigid ureteroscopes (non-steerable), but work by Marshall and later by Bagley, Huffman and Lyon firmly established flexible steerable instruments as an improvement, although with limitations. The term Retrograde Intra-renal Surgery (RIRS) was coined to denote this ability to inspect and work in all the calices of the kidney through the ureteral lumen. With the development of newer generation of flexible ureteroscopes with better optics, greater deflecting ability and smaller outer diameters, there has been widespread exuberance towards including more complex and larger renal stones and other renal pathologies in the RIRS basket. This symposium of articles by leaders in this field will, I hope, give the reader a clear and balanced view of the current status of this exciting and developing field.

Dr. Grasso's article provides an excellent overview of the development, present status of instrumentation, indications and possibilities of future development of flexible ureteroscopy. Additionally, the endourologist will need in his armamentarium a wide range of accessory instrumentation made by different companies each designed to achieve a particular objective. Dr. Monga makes some recommendations based on evidence he has been able to collect regarding one's choices in selecting “the right instruments in the right situation”.

It is well accepted that lower pole stones pose the greater problem towards elimination of fragments following SWL. Therefore, I thought that the study of different modalities of treating lower pole stones of different sizes (< 1 cm and 1−2 cm) would be informative to our readers. Dr. Shah has made an excellent critical review of various articles published in managing lower calyceal stones < 1 cm with flexible ureteroscopy. Holding a contrary view, Dr. Chaussy, one of the innovators of SWL has given his expert opinion based on his very considerable experience in defense of lithotripsy for same size stones. Over the last decade, stone clearance following SWL for larger (1-2 cm) lower pole stones has been unacceptably poor and this has resulted in widespread preference for PCNL for such stones. While stressing on the inherent complications of PCNL, Dr. Gross has shown safety and superiority of treating larger (1-2 cm) lower pole stones with RIRS. In my article, I have reviewed the role of PCNL for lower pole stones 1-2 cm in size, laying special stress on the newer developments in the field such as access, number of tracts and drainage. The literature review would suggest that mild increase in morbidity in PCNL is compensated by distinctly superior clearance rates. Pushing the envelope further, Dr. Mariano in his original article has detailed his technique of RIRS for renal stones > 2 cm in size. The reader will find the detailed description of the tips and tricks-of-the-trade helpful.

Stones form the most common indication for RIRS yet, the technique has been extended for treatment of pelvi-ureteric junction obstruction and upper tract transitional cell carcinoma (TCC) management. Dr. Desai's article deals with this aspect. Finally Dr. Mandhani has written a thought-provoking article after a review of the present literature on the advisability (or lack) of every endourologist having a flexible ureteroscope in his armamentarium. In conclusion, he argues that “patients who actually need flexible ureteroscopy should be referred to larger centers where this facility is available.”

It is my opinion that RIRS though here to stay, continues to evolve through improvements in instrumentation. The recent introduction of the prototype digital ureteroscope with “C-MOS chip on the tip” and light provided by twin LEDs, introduces the hope of a more durable, more steerable ureteroscope with significantly improved vision. Further innovations may one day achieve the goal of a sturdy yet cheap flexible ureteroscope that lasts over a 100 cases.

This symposium has brought one exceedingly important issue to light. There is at present a serious lack of Level 1 or level 2 evidence-based articles to support various claims of superiority of one or the other technique, namely RIRS, PCNL and SWL, in a given situation. Therefore, where evidence-based guidelines are missing, the urologist would have to make the wisest choice for his patients depending upon patients' preferences, his expertise, armamentarium available and cost considerations.

